# Incidental Detection of Glutamate Formiminotransferase Deficiency Through Newborn Screening in a Clinically Asymptomatic Infant: Molecular Findings and Counseling Considerations

**DOI:** 10.7759/cureus.105567

**Published:** 2026-03-20

**Authors:** Saja Baheer Abdulwahhab, Mohab Ben Omran, Osama Y A Aldirbashi

**Affiliations:** 1 Genetics, Sidra Medicine, Doha, QAT; 2 Research, Sidra Medicine, Doha, QAT; 3 Lab Medicine and Pathology, Hamad Medical Corporation, Doha, QAT

**Keywords:** formiminoglutamic aciduria, ftcd deficiency, genotype–phenotype correlation, inborn errors of metabolism, newborn screening

## Abstract

We report a seven-week-old male infant, born to consanguineous (double cousin) parents, incidentally through routine newborn screening, following an abnormal acylcarnitine profile caused by elevated formiminoglutamate (FIGLU), with subsequent biochemical confirmation of glutamate formiminotransferase cyclodeaminase (FTCD) deficiency. The infant was born at 37 weeks' gestation via spontaneous vaginal delivery, was vigorous at birth, and required no resuscitation. He remained clinically asymptomatic at the time of metabolic and genetic evaluation. Plasma acylcarnitine analysis demonstrated elevated formiminoglutamate (FIGLU) at a mass-to-charge ratio (m/z) of 287. Urine organic acid analysis revealed increased hydantoin-5-propionic acid, consistent with FTCD deficiency. Whole-genome sequencing identified a homozygous in-frame deletion in the FTCD gene, c.754_756del (p.Glu252del), inherited from both parents. This variant affects a conserved region of the bifunctional FTCD enzyme, which plays a critical role in histidine degradation and folate-dependent one-carbon metabolism. FTCD deficiency is an autosomal recessive disorder historically associated with megaloblastic anemia and mild neurodevelopmental delay. However, data from newborn screening programs increasingly demonstrate a spectrum of presentations, including individuals with isolated biochemical abnormalities and normal growth and neurodevelopment. In this case, the infant exhibited no clinical evidence of anemia, neurologic impairment, or failure to thrive. Complete blood count, folate, and vitamin B12 levels were within normal limits. Family history was notable for anemia of unknown etiology in a maternal aunt and grandmother, and breast cancer in a paternal aunt. This was the first child of the couple, with no prior affected siblings. This case reinforces that FTCD deficiency, while biochemically detectable, often follows a benign clinical course when identified presymptomatically. It highlights the expanding phenotypic spectrum of FTCD deficiency and underscores the importance of thoughtful biochemical follow-up, genotype-phenotype correlation, and tailored genetic counseling in the era of expanded newborn screening.

## Introduction

Glutamate formiminotransferase cyclodeaminase (FTCD) deficiency (Online Mendelian Inheritance in Man (OMIM) #229100) is a rare inborn error of metabolism affecting the final steps of histidine degradation and its connection to folate-dependent one-carbon metabolism. The FTCD gene encodes a bifunctional enzyme composed of glutamate formiminotransferase and cyclodeaminase domains, which together catalyze the conversion of formiminoglutamate (FIGLU) to glutamate through the transfer of the formimino group to tetrahydrofolate, thereby linking histidine catabolism to folate metabolism [1,2].

Loss of FTCD activity results in the accumulation of FIGLU and upstream intermediates. Biochemically, elevated FIGLU can be detected in plasma by tandem mass spectrometry (MS/MS) and in urine by gas chromatography-mass spectrometry (GC-MS), often accompanied by increased hydantoin-5-propionic acid, reflecting upstream blockade in histidine degradation [3]. Although FTCD deficiency was historically identified through targeted metabolic testing, it is now most frequently detected incidentally through expanded newborn screening (NBS) programs. In this context, FIGLU (mass-to-charge ratio (m/z) 287) may interfere with butyrylcarnitine (C4; m/z 288) quantification during MS/MS analysis, triggering false-positive screens for short-chain acylcarnitine disorders [4].

Prior to the implementation of expanded newborn screening, FTCD deficiency was classically associated with variable neurodevelopmental delay, megaloblastic anemia, and folate deficiency, based largely on clinically ascertained cases [3]. However, more recent newborn screening-based cohorts have demonstrated that the majority of affected infants remain clinically asymptomatic or exhibit isolated biochemical abnormalities without hematologic or neurodevelopmental impairment [5]. These observations suggest reduced penetrance and variable expressivity of FTCD deficiency, particularly when identified presymptomatically. Early detection through newborn screening provides an opportunity to better define the natural history of the condition, refine genotype-phenotype correlations, and guide evidence-based clinical follow-up and genetic counseling [6]. FTCD deficiency is considered a rare metabolic disorder, and its increasing identification through newborn screening highlights the importance of understanding its clinical spectrum and natural history.

## Case presentation

A seven-week-old male infant was referred following an abnormal newborn screening result reported in the early neonatal period, and confirmatory biochemical and genetic testing were completed within the first weeks of life, suggestive of a short-chain acylcarnitine disorder, with subsequent biochemical evaluation revealing FTCD deficiency. Confirmatory plasma acylcarnitine profiling demonstrated elevated FIGLU, and urine organic acid analysis by gas chromatography-mass spectrometry confirmed increased hydantoin-5-propionic acid and FIGLU.

He was born at 37 weeks’ gestation with a birth weight of 3.1 kg to healthy double-cousin parents. The perinatal course was unremarkable, and early growth and developmental milestones were age-appropriate.

Complete blood count results were within normal limits. Serum folate and vitamin B12 levels were normal. Physical examination revealed no dysmorphic features or hepatosplenomegaly. At the time of evaluation (7 weeks of age), the infant demonstrated appropriate growth and development. He was feeding well, had normal tone and activity, and showed age-appropriate responsiveness. Growth parameters were within expected ranges for age. Repeat laboratory evaluation, including complete blood count, remained within normal limits.

Whole-genome sequencing identified a homozygous likely pathogenic in-frame deletion (c.754_756del; p.Glu252del) in the FTCD gene. Both parents were confirmed heterozygous carriers, consistent with autosomal recessive inheritance.

The identified variant represents a rare in-frame deletion that is absent from population databases, including the Genome Aggregation Database (gnomAD) [7] and the Exome Aggregation Consortium (ExAC) [8]. The deletion affects a conserved residue within the formiminotransferase domain and is consistent with the observed biochemical phenotype. Segregation analysis demonstrated appropriate inheritance within the family. Based on combined molecular, biochemical, and segregation evidence, the variant meets ACMG/AMP criteria for likely pathogenicity. PM2 is supported by absence from population databases (gnomAD and ExAC), PM3 by detection in the homozygous state in a patient with a consistent biochemical phenotype and confirmed parental carrier status, PP3 by in silico predictions supporting a deleterious effect on a conserved residue, and PP4 by the highly specific biochemical phenotype of elevated FIGLU and hydantoin-5-propionic acid.

The family history was notable for parental consanguinity. A maternal aunt and grandmother had a history of anemia of unknown etiology. A paternal aunt had a history of breast cancer. The father was otherwise healthy with a history of an uncomplicated sleeve gastrectomy. There was no known family history of metabolic or hematologic disorders.

**Figure 1:**
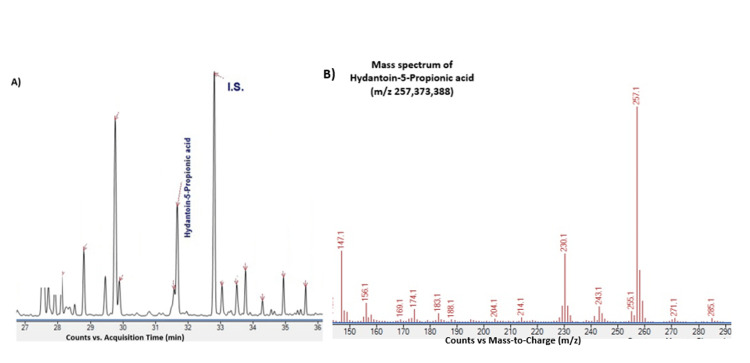
Urine organic acid analysis confirming hydantoin-5-propionic acid in glutamate formiminotransferase cyclodeaminase deficiency (A) Total ion chromatogram (TIC) demonstrating a prominent peak corresponding to hydantoin-5-propionic acid. (B) Mass spectrum obtained at the corresponding retention time showing characteristic fragment ions (mass-to-charge ratio (m/z) 257, 373, and 388), confirming the identity of hydantoin-5-propionic acid. Abbreviations: TIC, total ion chromatogram; m/z, mass-to-charge ratio

**Figure 2:**
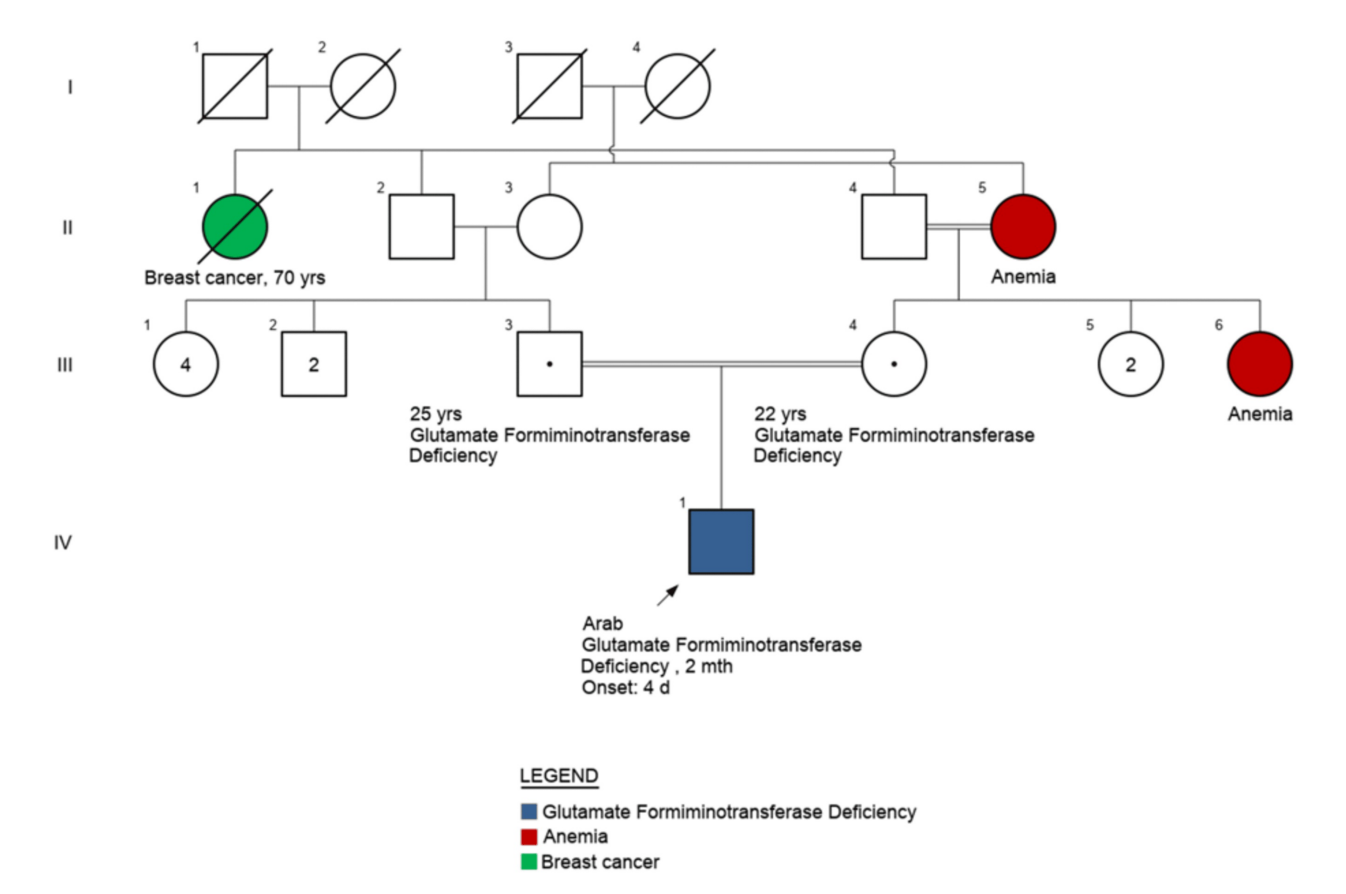
Family pedigree demonstrating autosomal recessive inheritance of glutamate formiminotransferase cyclodeaminase deficiency Squares represent males, circles represent females, and filled symbols indicate affected individuals. Anemia is indicated where reported in family members.

Urine organic acid findings are illustrated in Figure 1. The family pedigree demonstrating autosomal recessive inheritance is shown in Figure 2.

## Discussion

Published case series and newborn screening-based cohorts demonstrate a broad phenotypic spectrum in FTCD deficiency. Early clinically ascertained cases, described prior to the implementation of newborn screening, reported developmental delay, megaloblastic anemia, folate abnormalities, and marked FIGLU excretion, contributing to the perception of FTCD deficiency as a symptomatic metabolic disorder [3,9-12].

In contrast, cohorts identified through newborn screening have shown that the majority of individuals with FTCD deficiency remain clinically asymptomatic, exhibiting isolated biochemical abnormalities without neurodevelopmental impairment or hematologic manifestations [5]. Longitudinal follow-up data demonstrate normal growth and development in most screen-detected individuals, supporting reduced penetrance and variable expressivity [5].

Biochemically, recognition of FIGLU interference with butyrylcarnitine (C4) quantification during tandem mass spectrometry explains why FTCD deficiency is often detected incidentally through newborn screening panels designed for short-chain acylcarnitine disorders [4]. Awareness of this analytical overlap is essential to prevent misinterpretation of screening results and unnecessary diagnostic escalation.

Molecular characterization has demonstrated substantial allelic heterogeneity within the FTCD gene [2,6]. Reported FTCD variants include missense, nonsense, splice-site, and small in-frame deletions distributed across both functional domains of the enzyme. To date, no clear genotype-phenotype correlation has been established, as individuals harboring different variant types, including those predicted to significantly affect protein structure, may present with either symptomatic disease or isolated biochemical abnormalities. This variability highlights the complexity of predicting clinical outcomes based solely on molecular findings. Variant spectrum analyses indicate that both missense and truncating variants may result in biochemical phenotypes of variable clinical expression, complicating genotype-phenotype correlations [6]. Structural studies of the bifunctional FTCD enzyme further support the biological plausibility of phenotypic variability by illustrating the complex organization of the enzyme and its integration into folate-dependent one-carbon metabolism [13].

The present case aligns with the emerging newborn screening-detected phenotype, demonstrating biochemical confirmation of FTCD deficiency with preserved clinical health, normal hematologic indices, and appropriate early development. This presentation is consistent with contemporary newborn screening cohorts demonstrating a largely benign or minimally expressive clinical course [5].

The identification of a homozygous in-frame FTCD deletion in a clinically asymptomatic infant highlights the evolving challenges of genomic interpretation in the newborn screening era. While historically described cases emphasized symptomatic disease [3,9-12], expanded newborn screening has revealed a broader phenotypic continuum [5].

Allelic spectrum studies confirm genetic heterogeneity within FTCD [6], and molecular findings alone do not reliably predict clinical severity. Therefore, interpretation of pathogenic or likely pathogenic variants requires integration of biochemical confirmation, clinical evaluation, and longitudinal follow-up.

Counseling families requires clarification that FTCD deficiency is frequently identified incidentally due to FIGLU interference with C4 measurement in tandem mass spectrometry [4], rather than as a primary targeted screening disorder. Explaining this mechanism reduces unnecessary anxiety and improves understanding of the diagnosis. Given the autosomal recessive inheritance pattern, recurrence risk remains 25% for future pregnancies; however, the predominantly benign clinical course observed in newborn screening-detected individuals supports a conservative management approach focused on surveillance [5]. In line with this approach, a structured follow-up plan has been implemented for this patient. This includes periodic clinical assessments with monitoring of growth, developmental milestones, and hematologic parameters. At present, no specific treatment is required, given the absence of clinical symptoms, anemia, or folate deficiency.

## Conclusions

This case illustrates the evolving clinical landscape of FTCD deficiency in the era of expanded newborn screening. Identification of a homozygous in-frame FTCD deletion with biochemical abnormalities but no clinical manifestations reinforces the need for individualized follow-up and careful counseling. Our findings contribute to the growing evidence that many individuals with FTCD deficiency experience a benign clinical course and underscore the importance of longitudinal data to inform management guidelines.
